# The Mitochondrial Citrate Carrier SLC25A1/CIC and the Fundamental Role of Citrate in Cancer, Inflammation and Beyond

**DOI:** 10.3390/biom11020141

**Published:** 2021-01-22

**Authors:** Rami Mosaoa, Anna Kasprzyk-Pawelec, Harvey R. Fernandez, Maria Laura Avantaggiati

**Affiliations:** 1 Department of Oncology, Georgetown University Medical Center, Washington, DC 20057, USA; rmm84@georgetown.edu (R.M.); ak1801@georgetown.edu (A.K.-P.); 2 Department of Biochemistry, Faculty of Science, King Abdulaziz University, Jeddah 21589, Saudi Arabia; 3 Department of Neuroscience, Georgetown University Medical Center, Washington, DC 20057, USA; Harvey.Fernandez@georgetown.edu

**Keywords:** SLC25A1, CIC, CTP, citrate, mitochondria, cancer, metabolism, inflammation, diabetes, 22.q11.2, NAFLD/NASH

## Abstract

The mitochondrial citrate/isocitrate carrier, CIC, has been shown to play an important role in a growing list of human diseases. CIC belongs to a large family of nuclear-encoded mitochondrial transporters that serve the fundamental function of allowing the transit of ions and metabolites through the impermeable mitochondrial membrane. Citrate is central to mitochondrial metabolism and respiration and plays fundamental activities in the cytosol, serving as a metabolic substrate, an allosteric enzymatic regulator and, as the source of Acetyl-Coenzyme A, also as an epigenetic modifier. In this review, we highlight the complexity of the mechanisms of action of this transporter, describing its involvement in human diseases and the therapeutic opportunities for targeting its activity in several pathological conditions.

## 1. Introduction

**Figure 1:**
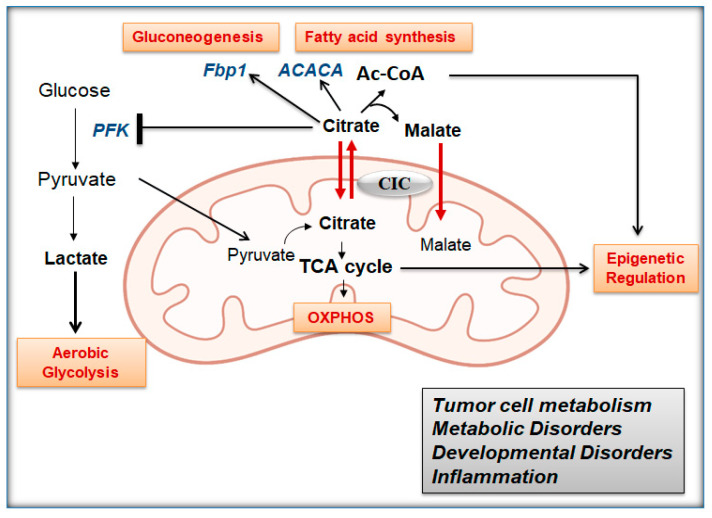
Preview of the activities of citrate/isocitrate carrier (CIC) (also see text for explanation).

The mitochondrial citrate carrier SLC25A1, also known as citrate transporter protein (CTP) or citrate/isocitrate carrier (CIC), is a mitochondrial membrane transporter encoded in the nucleus by the *SLC25A1* gene located on chromosome 22q11.2. Historically, CIC was first purified and reconstituted by Palmieri [1,2,3] and subsequently by Kaplan [4], and as of today, it is the only known human mitochondrial transporter for citrate, which renders its activity of paramount importance. The known function of CIC consists of promoting the export of citrate or isocitrate from the mitochondria into the cytosol in exchange for malate (Figure 1). Cytosolic citrate has several fundamental functions, on one side providing the source of Acetyl-Coenzyme A (Ac-CoA) for fatty acids and sterol biosynthesis, and on the other, serving as an allosteric regulator of enzymes that control glycolysis, lipogenesis and gluconeogenesis [5,6]. The activity of phosphofructokinase (PFK), a glycolytic enzyme, is inhibited by citrate binding, while 1,6-bisphosphatase (Fbp1) and Acetyl-CoA Carboxylase Alpha (ACACA), necessary for gluconeogenesis and lipid synthesis, respectively, are activated by citrate [7,8,9]. We and others have shown that through reverse import activity, CIC can also promote the entry of cytosolic citrate into the mitochondria, stimulating the tricarboxylic acid (TCA) cycle and oxidative phosphorylation (OXPHOS) and maintaining redox balance through the generation of NADPH, all activities that are important for the expansion of cancer stem/initiating cells and for the anchorage-independent growth of tumor cells [10,11]. In addition, CIC is likely a mediator of the “mitochondrial-to-nucleus-cross-talk” through which metabolic adjustments originating in the mitochondria are transmitted to the nucleus and reshape the transcription program via epigenetic regulation [12]. This activity of CIC, albeit still understudied, stems from its ability to provide Ac-CoA for acetylation reactions, to enhance the availability of TCA cycle intermediates that act as epigenetic regulators, particularly succinate, fumarate and alpha-ketoglutarate (α-KG), and to prevent the accumulation of L- and D-2-hydroxyglutaric acids, two oncometabolites that inhibit histone demethylases and are abnormally elevated when CIC activity is impaired [5].

Through all these complex functions, CIC sits at the center of the metabolic landscape of cells, serving a key role for the generation and optimal utilization of resources needed to meet the energetic demand of tissues under physiological conditions. It is, therefore, not surprising that loss of CIC is pathogenic, and in fact, mutations or mono-allelic deletions of the *SLC25A1* gene have been linked to a complex and heterogeneous spectrum of developmental diseases. Alterations of CIC activity also occur in autoimmune disorders such as rheumatoid arthritis and Bechet’s disease and in Down syndrome [13,14]. Furthermore, amplifications of the *SLC25A1* gene or enhanced transcription rates are a hallmark of several cancer types as well as of metabolic disorders [15,16]. Here, we will dissect the complexity of the mechanisms of action of CIC in human diseases and rationalize the advantages of therapeutic targeting of its activities.

## 2. A Brief History of CIC Inhibitor Compounds

**Figure 2:**
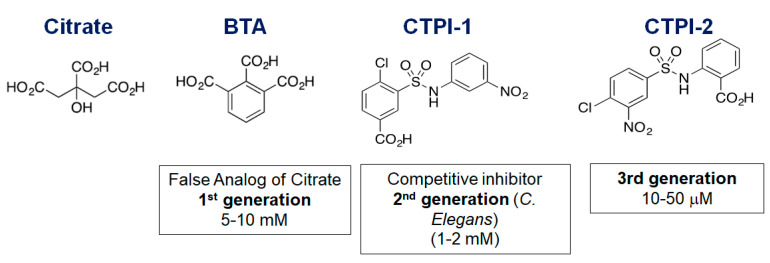
Comparison of the structure of CIC inhibitors. The in vivo IC50 is indicated for each of the compounds.

Given the involvement of CIC and of the lipogenic pathway in cancer, the development of CIC inhibitors has been at the cornerstone of the field, but not without challenges (Figure 2). The first-generation inhibitor benzenetricarboxylate (BTA) is a false and non-cleavable analog of citrate that had been widely employed in in vitro assays on reconstituted liposomes to block CIC transport activity [1,2,3,4].

In vivo, BTA requires very high concentrations (5 mM) and is also potentially able to interfere with the activity of other citrate-binding proteins. A second inhibitor, CTPI-1, or 4-Chloro-3-[[(3-nitrophenyl)amino]sulfonyl]-benzoic acid (CNASB), was discovered by Kaplan’s group based on a homology model of *Caenorhabditis elegans* CIC. CTPI-1 is the first competitive inhibitor and was shown to interact with key residues involved in citrate binding [17]. Among these, Arg181 in yeast protein is replaced by Lys190 in human protein. Given that this residue is in the citrate binding pocket, the affinity of CTPI-1 for the human protein is not ideal, with an experimental *K*_D_ of 60 µM, as determined with surface plasmon resonance (SPR) [10]. Accordingly, CTPI-1 also requires very high concentrations for in vivo activity (1–2 mM). Based on this, our group undertook several approaches to optimize compounds specific for the human CIC protein, employing an in silico homology model, docking experiments and searching similar compounds in available databases, followed by SPR to characterize the interaction of purified CIC with identified candidates [10]. By exchanging the position of the chlorine atom, we identified a compound (CTPI-2, or 2-(4-Chloro-3-nitro-benzenesulfonylamino)-benzoic acid) that now exhibits an experimental *K*_D_ between 1 and 3.5 µM, a 20-fold improvement in binding activity relative to CTPI-1, and inhibits citrate transport and tumor proliferation at significantly lower doses (10–50 µM).

Interestingly, all of the CIC inhibitors are relatively insoluble (unpublished observations). We believe that the relative insolubility of these drugs is important for their interaction with—and transport through—the cytoplasmic and mitochondrial membranes and, thus, may be an intrinsic characteristic of this class of agents. As discussed in this review, CTPI-2 and other CIC inhibitors belong to a novel class of promising therapeutics. 

## 3. Regulation of CIC Expression Levels: Hints on Its Biological Functions

**Figure 3:**
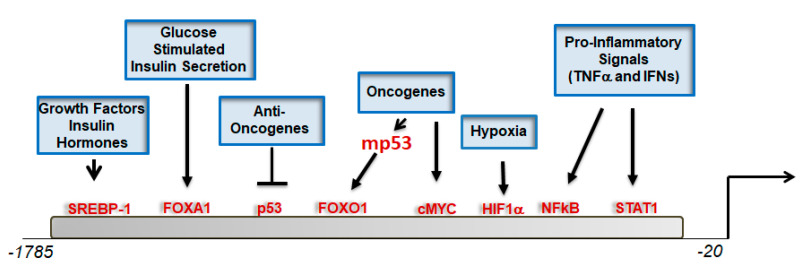
Schematic representation of the CIC promoter and transcription factors’ binding sites (TFBSs) identified in various studies. The positions of these TFBSs are representative and do not reflect the actual position in the promoter.

There are several ways through which CIC activity can be engaged in cells under physiological and pathological conditions, and these often involve transcriptional regulation. Early work performed by the Palmieri group showed that the transcription rate of the CIC promoter is under the control of the master regulator of lipid anabolic pathways, sterol regulatory element-binding factor 1 (SREBP1) [18] (Figure 3), and of Forkhead Box A1 (FOXA1), which, via CIC, induces glucose-stimulated insulin secretion [19].

The tumor suppressor p53 also interacts with the CIC promoter [20]. However, while wild-type p53 suppresses transcription, tumor-associated mutant(s) forms of p53 do not bind to the promoter directly but are recruited therein through interaction with the transcription factor Forkhead Box O1 (FOXO1), which has very important activities in the regulation of insulin signaling, gluconeogenesis and glycogenolysis. Binding sites for the p65 subunit of nuclear factor kappa B (NFκB) and for signal transducer and activator of transcription 1 (STAT1) promote CIC transcription in response to tumor necrosis factor alpha (TNFα) and interferon gamma (IFNγ), two important mediators of inflammation [21]. Further, the transcription factors Myc, hypoxia-inducible factor 1 alpha (HIF1a) and peroxisome proliferator-activated receptor gamma (PPARγ) also interact with the CIC promoter [22]. These mechanisms of activation anticipated fundamental activities of CIC in the metabolism and inflammation and suggested that this protein might provide a link between oncogenic pathways and the glucose and lipid tumor cell metabolism, as discussed below.

## 4. CIC Activity in Glucose and Lipid Metabolism: Implications for Metabolic Diseases

**Figure 4:**
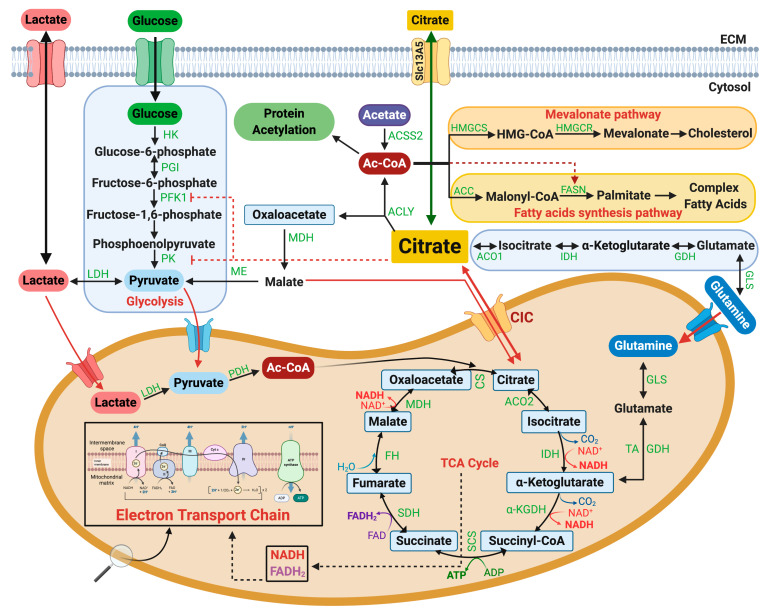
Pathways to the generation of cytosolic and mitochondrial citrate and its utilization. Glucose-derived citrate is obtained through the conversion of glucose to glucose-6-phosphate, which, with a series of enzymatic reactions, is then converted to pyruvate. Pyruvate is reduced to lactate via lactate dehydrogenase (LDH) or, alternatively, transported into mitochondria to produce Acetyl-Coenzyme A (Ac-CoA) via pyruvate dehydrogenase (PDH). Citrate synthase then catalyzes the condensation of acetyl-CoA with oxaloacetate to yield citrate that is exported in the cytosol by CIC. Lactate can also enter the mitochondria and be converted to pyruvate by a mitochondrial lactate dehydrogenase (mtLDH) regenerating citrate. Mitochondrial citrate and, to a lesser extent, lactate fuel the tricarboxylic acid (TCA) cycle and the electron transport chain (ETC). Citrate can also be uptaken from the extracellular space and transported to the cytosol via SLC13A5. In the cytosol, citrate provides Ac-CoA via ATP citrate lyase (ACLY) for protein acetylation and can enter the mevalonate pathway for cholesterol biosynthesis mediated by hydroxymethylglutaryl-CoA synthase (HMGCS) and hydroxy-3-methylglutaryl-CoA reductase (HMGCR) and the fatty acid synthetic pathway via acetyl-CoA carboxylase (ACC) and fatty acid synthase (FASN). Cytosolic Ac-CoA can be also generated by acetyl-CoA synthetase 2 (ACSS2) which converts acetate derived from deacetylation reactions to acetyl-CoA. Cytosolic citrate inhibits phosphofructokinase 1 (PFK1) and pyruvate kinase (PK), thus playing an active role in controlling glycolytic flux. An alternative source of mitochondrial or cytosolic citrate is supplied by reductive carboxylation of alpha-ketoglutarate to isocitrate, mediated in the cytosol by isocitrate dehydrogenase 1 (IDH1) and in the mitochondria by IDH2. Additional abbreviations: HK—hexokinase; G6PD—glucose-6-phosphate dehydrogenase; 6PGL—6-phosphogluconolactonase; 6PGD—6-phosphogluconate dehydrogenase; Rpi—ribose-5-phosphate isomerase; PGI—phosphoglucose isomerase; ME—malic enzyme; MDH—malate dehydrogenase; CS—citrate synthase; ACO2—aconitase 2; IDH—isocitrate dehydrogenase; α-KGDH—α-Ketoglutarate dehydrogenase; SCS—succinyl coenzyme A synthetase; SDH—succinate dehydrogenase; FH—fumarase; ACO1—aconitase 1; GHD—glutamate dehydrogenase; GLS—glutaminase.

Citrate is at the cross-roads of multiple metabolic pathways (depicted in Figure 4) and it is an indispensable carbon source in both the mitochondria and the cytosol. When glucose is abundant, the majority of mitochondrial and cytosolic citrate comes from the oxidative decarboxylation of pyruvate to form Ac-CoA via the pyruvate dehydrogenase complex (PDC) and subsequent condensation of Ac-CoA with oxaloacetate to form citrate and Co-A. These reactions, coupled with the activity of pyruvate carboxylase (PC) that provides mitochondrial oxaloacetate, promote the TCA cycle and the generation of NAD+, NADH and FADH2 for the electron transport chain (ETC), also allowing for CIC-mediated transport of citrate. Through the export activity, CIC is proposed to be essential for lipid and cholesterol synthesis.

**Figure 5:**
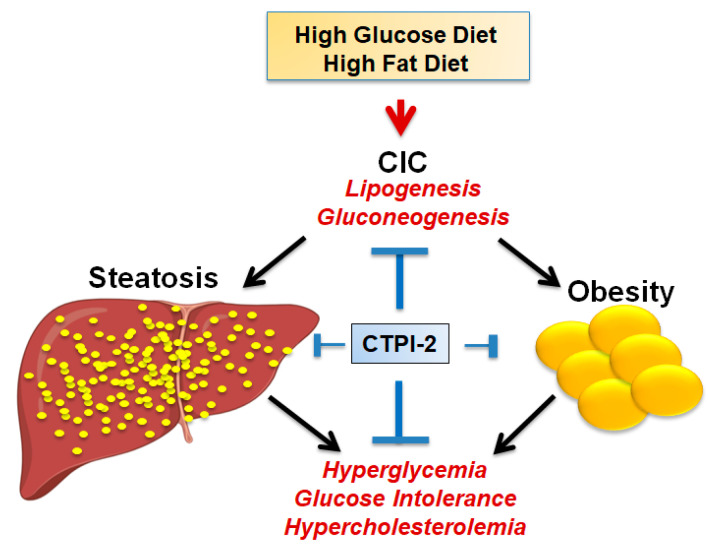
Schematic representation of some of the salient effects of CTPI-2 in the non-alcoholic fatty liver disease/non-alcoholic steatohepatitis (NAFLD/NASH) model.

In agreement with this idea, the activity of CTPI-2 and of a liver-targeted CIC knockout was recently studied in a well-established murine model of diet-induced Non-alcoholic fatty liver disease (NAFLD) and non-alcoholic steatohepatitis (NASH), the diet-induced obesity (DIO) mouse model [16]. When these mice are fed a diet enriched in starch and lard, they develop severe obesity, accompanied by hypercholesterolemia, hypertriglyceridemia, glucose intolerance, hyperglycemia and fatty liver disease, which, with time, can progress to steatohepatitis and eventually to hepatocellular carcinoma. CTPI-2 not only reverts or prevents steatosis and markedly reduces obesity, depending upon the administration schedule, but also normalizes cholesterol and triglyceride levels as well as hyperglycemia and glucose intolerance (Figure 5). Indeed, normalization of glucose metabolism is the most significant outcome of CTPI-2 treatment. A global metabolomic analysis provided strong evidence that gluconeogenesis, a key contributor to the levels of circulating glucose, is a major target pathway inhibited by CTPI-2, together with the expected inhibition of fatty acid synthesis sustained by a reduction in the Ac-CoA pool.

Another important clue derived from studying the effects of CTPI-2 in vivo is that the expression levels of CIC are regulated systemically by the levels of circulating glucose, being repressed by a low-glucose diet and strongly induced by a calorie equivalent, high-glucose regimen that leads to hyperglycemia. Together with the finding that CTPI-2 reverts glucose intolerance and insulin resistance, these results raise the fascinating possibility that CTPI-2 and, in general, CIC inhibitors may act as glucose-mimetic agents. These results expand the potential applicability of this class of drugs to metabolic syndrome and diabetes, which have reached epidemic proportions worldwide and are a major cause of morbidity and mortality. Studies in this direction should be very exciting.

Interestingly however, the knockout of the *SLC25A1* gene targeted to the liver through an Albumin/Cre-regulated promoter (L-CIC-KO) only partially recapitulates the effects of CTPI-2. While CTPI-2 completely normalizes the biochemical and histological characteristics of DIO mice in the liver, adipose tissue and systemically, in the liver of the CIC-KO mice, steatosis is reduced but not completely blunted. There are two non-mutually exclusive possible explanations for this result. The first is that the beneficial effects of CTPI-2 rely upon inhibition of CIC systemically and not only in the liver but, at the very least, also in the adipose tissue, which is a key contributor to the metabolic alterations observed in NAFLD/NASH. These beneficial effects might also rely upon induction of weight loss by CTPI-2. Testing of this hypothesis will require the generation of additional mouse models harboring the *Slc25a1* gene knockout also in the adipose tissue. An alternative possibility is that when the knockout of CIC is imposed during embryogenesis and development, as in the case of the Albumin/Cre-regulated promoter for the induction of the L-CIC-KO, there is a strong selective pressure for compensation to loss of CIC.

## 5. Is CIC Rate Limiting for De Novo Lipid Synthesis?

As shown in Figure 4, there are at least three CIC-independent pathways for providing cytosolic citrate or the universal precursor for de novo fatty acid synthesis, Ac-CoA. Acyl-CoA synthetase short-chain family member 2, ACSS2, converts acetate derived from deacetylation reactions to acetyl-CoA. There is evidence that ACSS2 enriches the Ac-CoA pool, directing it to fatty acid and phospholipid synthesis in conditions of metabolic stress [23,24].

Several groups have also reported on the importance of plasma membrane citrate transporters (PMCTs), which uptake citrate from the extracellular space to enrich the cytosolic pool and display a tissue-specific pattern of expression. These transporters differ from CIC not only structurally, but also because they operate in a Na+- or K+-dependent manner. Interestingly, an alternatively spliced product of CIC itself, membrane CIC (mCIC), can serve this function, especially in the prostate [25,26]. The SLC13A5 transporter, the human counterpart of the *fly* gene *I’m not dead yet* *(INDY)*, is the most relevant PMCT as it is widely expressed in many tissues [27,28]. Extracellular citrate is derived from the liver and renal catabolism, and also through nutritional intake and bone remodeling, and is present in the plasma at very high—but homeostatically regulated—concentrations. It is possible that the activity of this transporter contributes to the cytosolic pool of citrate. Consistent with this idea, similarly to CIC, SLC13A5 is upregulated in diet-induced NAFLD/NASH and its inhibition prevents some of the pathological hallmarks of this disease [28]. Moreover, SLC13A5 is induced in DIO livers treated with CTPI-2, coinciding with a reduction in the concentration of serum citrate, thus suggesting that this protein can provide a mechanism of compensation when CIC is inhibited [16]. With this in mind, combinatorial therapy with CTPI-2 and SLC13A5 inhibitors (e.g., gluconate [29]) is likely to be more effective than treatment with either agent alone in NAFLD/NASH.

Last but not least, an important mode of replenishing the cytosolic citrate pool has been described by DeBerardinis’ group and occurs in tumor cells with defective mitochondria that cannot derive citrate from glucose via mitochondrial oxidative metabolism [30]. In cells with defective respiratory complex activities, glucose is diverted towards lactic acid production and glutamine provides the source of citrate. This pathway involves the reductive carboxylation of glutamine-derived α-KG to citrate via the action of isocitrate dehydrogenase 1 (IDH1) in the cytosol or of IDH2 in the mitochondria (Figure 4). This alternative source of citrate comes into play not only as an adjustment to defective mitochondrial oxidative capacity but also during hypoxia and in response to stress signals that generally alter the ratio between α-KG and citrate [31,32]. Recent work, also from DeBerardinis’ group, demonstrates that reductive carboxylation provides a source for lipid synthesis in cancer cells when CIC is inactivated via RNA interference, pointing to reductive carboxylation as a potential mechanism of compensation for CIC deficiency as well [33]. Furthermore, in children affected by DiGeorge syndrome sustained by hemizygous loss of the 22q11.2 chromosome, a CIC loss-of-function metabolic/mitochondrial signature was identified but was hallmarked by an increase in reductive carboxylation and enhanced α-KG levels associated with increased concentrations of 2-hydroxyglutaric acid, cholesterol and fatty acids—highly indicative of compensation through this pathway [34].

Based on these lines of evidence, it seems unlikely that CIC is rate-limiting for lipid and sterol biosynthesis or for other cytosolic functions related to citrate in all situations. It is possible that the opportunities for compensation of CIC activity through the above-mentioned pathways are tissue-specific, are dictated by the impending selective pressure for compensation (embryogenesis/development vs. post-natal life) and differ depending upon the nutritional/metabolic environment and the duration of such inhibition (chronic vs. acute). Whether the lipid synthetic pathway is altered in children harboring loss-of-function mutations of the *SLC25A1* gene and correlates with the severity of the clinical manifestations is a very important question that is still unanswered.

## 6. Pro-Oncogenic Activities of CIC, the Reversal of the Warburg Effect and the Phenomenon of Metabolic Addiction

For many years, the field of tumor cell metabolism has been pervaded by the assumption that mitochondria are dysfunctional in cancer cells and that tumors depend upon glycolysis for growth. The Warburg effect, described by Otto Warburg in 1920, is based upon the observation that cancer cells avidly uptake glucose and direct it towards fermentation to lactate in the cytosol, even at high oxygen concentration, rather than to complete oxidation in the mitochondria, a much more efficient pathway for ATP generation. This is, of course, the basis for positron emission tomography, or PET scans, However, two important concepts have emerged in recent years. First, the lactate generated through glycolysis can fuel the TCA cycle and mitochondrial oxidative phosphorylation [35], and therefore, glycolysis is not mutually exclusive with OXPHOS as these pathways can operate simultaneously in tumor cells. Second, and equally importantly, tumors contain metabolically heterogeneous populations of cells that utilize different branches of the metabolism depending upon proliferation rates as well as upon the “geographical” location of cells within tumors which, due to the irregularity of the tumor vasculature, are exposed to hypoxia and have less access to nutrients. This heterogeneity has not only been shown in vitro but also in patients affected by lung cancer and glioblastoma [36,37].

Several lines of evidence have demonstrated that CIC supports the outgrowth of cancer cells, yet the pro-proliferative activity of CIC relies upon promotion of OXPHOS and blunting of glycolysis, presumably due to the negative feedback loop that cytosolic citrate provided by CIC imposes on PFK [10,15,33]. Indeed, a consequence of CIC inhibition, genetically or pharmacologically, is impairment of mitochondrial oxidative capacity, reduction in mitochondrial-derived ATP output, accumulation of reactive oxygen species (ROS) and reduced abundance of TCA cycle intermediates. How CIC influences OXPHOS is still not entirely clear, but such regulation might occur at least in part due to promotion of malate entry into the mitochondria, in turn leading to increased TCA cycle flux and generation of adequate ratios of reducing equivalents, including NADH/NAD+, for the electron transport chain. In addition, the reverse import activity of cytosolic citrate via CIC can also promote mitochondrial oxidative metabolism through similar mechanisms.

**Figure 6:**
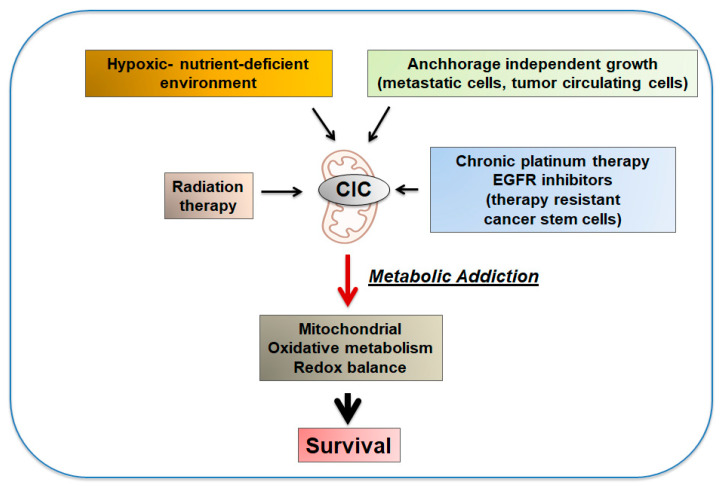
Involvement of CIC-dependent mitochondrial oxidative metabolism in adaptation to stress (see text for explanation).

Oxidative phosphorylation also provides a mechanism of resistance and adaptation to various stress conditions, as well as to chemotherapeutic agents and radiotherapy (Figure 6). In lung, prostate and glioblastoma cancer cells, CIC inhibition results in compromised mitochondrial oxidative capacity and oxidative stress and leads to increased sensitivity to radiation therapy [38,39]. Similarly, cancer cells resistant to platinum-derived agents or to Epidermal Growth Factor Receptor (EGFR), inhibitors develop an addiction to CIC-mediated promotion of mitochondrial respiration, and inhibition of CIC leads to synthetic lethality [10]. These therapy-resistant populations have characteristics of cancer stem cells and acquire markers of dormancy in a mitochondrial respiration-dependent manner. CIC allows these cells to survive therapeutic attacks in a paradoxically high energetic state. These results are in agreement with the idea that the cancer stem cell population provides a reservoir of cells left behind by conventional therapies and suggest that inhibition of CIC can be exploited as a therapeutic strategy to specifically target and eradicate therapy-resistant cells. Furthermore, CIC-dependent mitochondrial oxidative metabolism and redox balance provide a mechanism of adaptation and survival when tumor cells are challenged by limiting concentrations of glucose or must overcome addiction to the extracellular matrix and adapt to anchorage-independent growth [11,15], which is fundamental for the invasive and metastatic behavior of tumor cells.

In essence, these observations indicate that there is a therapeutic opportunity for targeting CIC activity, particularly in stress growth conditions and in cancer cell populations (e.g., cancer stem cells, therapy-resistant cells and metastatic, circulating tumor cells) that develop a strong dependency upon this protein and mitochondrial oxidative metabolism for survival. Although, as discussed above, there are many mechanisms that could compensate for CIC inhibition, especially because the metabolism of tumors is endowed with a high degree of heterogeneity and plasticity, cancer cells that have evolved to employ CIC for survival might develop a “metabolic addiction” to CIC and, thus, will not so quickly or easily surrender its activity. This phenomenon parallels the well-known oncogene addiction whereby tumors cells that have acquired oncogenic potential through the action of one driver oncogene remain dependent upon that oncogene to maintain their proliferative capacity, in spite of the accumulation of other complex genetic alterations. This is an attractive possibility that deserves further scrutiny.

## 7. CIC Inhibition Inhibits the Growth of Different Tumor Types

Given the large body of literature demonstrating a role of CIC in various malignancies, here, we summarize the results of these studies and we group them by tissue/tumor type.

CIC activity has been studied by several groups in breast cancer. CIC mRNA levels are generally elevated in breast cancer cells, particularly in triple-negative breast cancer cell lines (TNBC) [15]. Furthermore, CIC overexpression is associated with metastatic disease and poor patient prognosis [40]. In the MBA-231 TNBC cell line, which has high levels of CIC, the introduction of a CIC dominant negative mutant protein that competes with endogenous CIC or treatment with BTA or CTPI-1, leads to reduction in tumor size in vivo and in proliferation in vitro [15,41]. The effects of CIC inhibition were shown to be dependent upon changes in histone acetylation, mitochondrial dysfunction and ROS production. Targeting ACLY also effectively inhibits breast tumorigenesis [41].

In colorectal cancer (CRC), the expression levels of CIC were found to be upregulated by PPARγ coactivator 1α (PGC1α) and correlated with enhanced OXPHOS, TCA cycle flux and with de novo lipogenesis [42]. In this context, PGC1α promotes tumor growth and loss of PGC1α leads to a reduction in CIC and ACLY expression and to blunting of tumor growth, an effect recapitulated by inhibition of fatty acid synthesis. These studies reveal a strong connection between CIC and PGC1α and lead to the proposal that the activity of PGC1α on mitochondrial and lipid metabolism is mediated, at least in part, via CIC. Intriguingly, independent experiments in vivo where rats were injected with CRC cell lines revealed that a combination chemotherapy frequently used for the treatment of CRC (Irinotecan combined with 5-fluorouracil) has a prominent impact on adipose tissue, causing adipocytes to decrease in size [43]. This effect was correlated to reduced expression of proteins involved in fatty acid synthesis, including CIC, and in the esterification of fatty acids. This observation implies that CIC is also relevant to the loss of adipose tissue mass that occurs during chemotherapy.

CIC upregulation via PGC1α was also found in liver cancer cell lines (HCC) [44,45]. Higher citrate flux from mitochondria into the cytosol was reported in the hepatoma cell lines MH-3924A and Hepa-6 compared to normal liver cells, as well as in HEPG2 cells. Poolsri and colleagues showed that treatment of HepG2 or of another HCC cell line, HuH-7, with the CIC inhibitor CTPI-1 and with the SLC13A5 inhibitor (PMCTi) leads to significant reduction in cell viability [46]. However, this combination is non-toxic to primary normal human hepatocytes but, nevertheless, acts synergistically in promoting apoptosis, paralleled by inhibition of fatty acid biosynthesis compared to each inhibitor alone. This result is in agreement with the possibility that in the liver, combined inhibition of CIC and SLC13A5 provides a therapeutic benefit.

Recent studies have shown that in patients affected by papillary thyroid carcinoma (PTC), the long non-coding non-coding RNA for association with Brahma, (lncBRM), is significantly upregulated, correlating with poor overall survival [47]. lncBRM targets CIC activity via the microRNA miR-331-3p and promotes PTC cell proliferation, migration and invasion, an effect that is rescued by inhibiting CIC. These results suggest that also non-coding RNAs can regulate CIC expression or activity.

Reprogramming towards the citrate-mediated lipogenic pathway is a hallmark of prostate cancer. Recent studies have shown that prostate cancer cells exhibit elevated levels of citrate and display enhanced uptake of fatty acids, particularly at a metastatic stage [48,49,50]. Inhibition of cluster of differentiation 36 (CD36), which promotes fatty acid uptake, or of CIC, leads to suppression of cancer progression. As mentioned previously, CIC expression was also found to be increased in prostate cancer cells under conditions of cycling hypoxia/re-oxygenation stress, and its inhibition results in increased sensitivity to radiation therapy. These effects were connected to reduced mitochondrial oxidative capacity, generation of ROS and impaired DNA repair capacity [38].

Viewed together with the evidence discussed before (Section 6), these multiple studies highlight that CIC is essential for the growth and proliferation of different cancer types and underscore the potential importance of advancing CIC inhibitors to the clinical setting, where they are likely to be most effective in combinatorial therapies.

## 8. CIC and Citrate Are Important Mediators of Inflammation

Since the 19th century, inflammation has been considered a key promoter of many human diseases, including cancer, playing a role in as many as 20% of all tumors in humans [51,52]. As of today, there are several lines of evidence suggesting that CIC may fuel inflammation [53].

**Figure 7:**
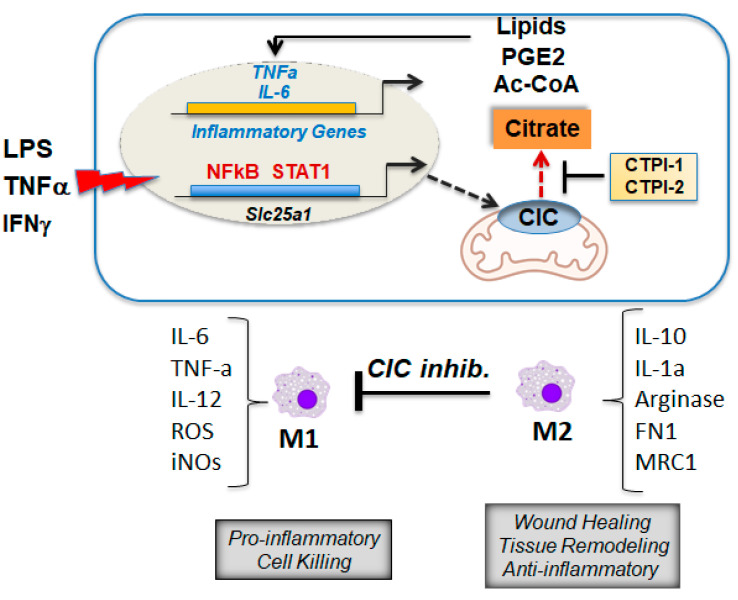
CIC induces a pro-inflammatory program in macrophages (see text for explanation). The transcription rate of the *SLC25A1* gene is induced in monocytes by lipopolysaccharides (LPS), tumor necrosis factor alpha (TNFα) and interferon gamma (IFNγ) via recruitment to the promoter of nuclear factor kappa B (NFkB) and signal transducer and activator of transcription 1 (STAT1). CIC induction in these situations leads not only to the expected increase in Ac-CoA but also to enhanced synthesis of prostaglandin E2 (PGE2) and inducible nitric oxide synthetase (iNOS). At the bottom of the figure, there is a simplistic representation of the macrophage populations depicted in the two opposite phenotypes, M1 and M2. In the NAFLD/NASH liver, CIC inhibition represses markers of the pro-inflammatory macrophage phenotype. Abbreviations that are not in the main text: reactive oxygen species, ROS; iNOS, inducible nitric oxide synthase; FN1, fibronectin 1; MRC1, Mannose Receptor C-Type 1.

CIC was shown to be induced in monocytes/macrophages by lipopolysaccharides (LPS) and in the U937 monocytic cell line by TNFα and IFNγ [21] (Figure 7). This activation occurs via NF-κB and STAT1 and leads to an increase in the cytosolic pool of citrate used for de novo fatty acid synthesis. Inhibition of CIC with CTPI-1 or with interfering RNAs leads to reduction in citrate export and depletion of pro-inflammatory prostaglandin E2 (PGE2), which is derived through Ac-CoA metabolism, and also of nitric oxide (NO), an important mediator of the inflammatory response. Subsequent studies showed that ACLY also promotes pro-inflammatory changes in macrophages through mechanisms similar to those shown for CIC [54]. Given that ACLY consumes citrate to provide Ac-CoA, the involvement of this protein underscores the importance of the lipid synthetic pathway in this reprogramming and, likely, also of epigenetic modifications induced via acetylation. The expression levels of CIC and ACLY were subsequently found to be increased in cytokine-stimulated natural killer (NK) cells [55], suggesting that similar activities of CIC may take place in other immune cell populations.

Based on the above mentioned studies, important targets of CIC pro-inflammatory activities are macrophages. This was later corroborated in murine models of NAFLD/NASH [16]. Inflammation is a major driver of pathology in this disease, eventually responsible for the evolution to irreversible fibrosis driven by remodeling of the extracellular matrix under the constant insults propelled by pro-inflammatory signals [56]. Macrophages and, particularly, the M1 population are at least in part responsible for these alterations [57]. M1 macrophages produce both pro-inflammatory and immuno-stimulatory cytokines, particularly interleukin (IL)-12, IL-6, IL-1α, TNF-α and IL-1β, and create an environment that is microbicidal in the context of the innate immune response and leads to cell death. The alternative M2 pathway of activation plays an important role in tissue repair and homeostasis. M2 macrophages produce IL-10, mitogens and fibronectin and deplete the environment of l-arginine via induction of Arginase-I, which is required for T cell functions. In patients with NASH, resident and recruited macrophages in the liver as well as recruited macrophages in the adipose tissue contribute to the production of local and systemic TNF-α and IL-6, two important mediators of inflammation. In the murine model of this disease, inhibition of CIC with CTPI-2 results in lower serum levels of IL-6, TNFα and monocyte chemoattractant protein 1 (MCP1), concomitant with an increase in cytoprotective IL-10 and IL-4 [16]. Moreover, CTPI-2 leads to reduced macrophage recruitment in the liver, but more prominently so in the adipose tissue, as well as suppression of markers of the M1 phenotype and attenuation of the levels of tissue-damaging cytokines, particularly iNOS and TNFα, while not affecting or moderately increasing markers of M2 activation [16]. These lines of evidence argue—but do not prove, yet—that CIC contributes to the polarization of macrophages towards a pro-inflammatory M1 phenotype and raise the important question as to whether such reprogramming occurs via CIC-induced epigenetic modifications. Given that inflammation is an important driver of oncogenesis, it is further possible that this activity of CIC also contributes to tumor proliferation.

On the more speculative side, while the finding that CIC is under the control of NFκB and STAT1 highlights its involvement as a mediator of inflammation, it also suggests that CIC might be connected to the innate anti-viral immune response, especially considering the prominent role of STAT1 in the regulation of this pathway. Interestingly, various citrate derivatives were shown to elicit anti-pathogen defenses providing protection not only from microbial but also from viral infections [58]. We posit that even though CIC has thus far been linked to cancer, developmental diseases and metabolic disorders, it is unlikely that the pro-inflammatory activities of CIC have been selected during evolution to sustain pathological conditions that often result in death. From an evolutionary point of view, this would make no sense. It is possible, instead, that there is a physiological role of CIC in the pro-inflammatory arm of the innate immune response and that such activity is aberrantly co-opted in disease states sustained by this protein.

## 9. Loss of CIC during Embryogenesis and Development: The L-and D-2HGA “Affair”

Alterations in the *SLC25A1* gene play a role in the pathogenesis of various developmental disorders, as recently reviewed by Palmieri and colleagues [5]. Heterozygous *SLC25A1* gene deletions are associated with congenital 22q11.2 microdeletion syndromes, namely Velo-Cardio-Facial and DiGeorge syndromes [59,60]. This is a group of disorders characterized by cleft palate, heart abnormalities, recurrent infections and autoimmunity, craniofacial and palate abnormalities and developmental and intellectual delay. Although only one copy of the *SLC25A1* gene is lost in 22q11.2 deletion syndrome, together with 30–40 other genes, a reduction in CIC levels in zebrafish (Danio Rerio) leads to abnormal morphant development in a dose-dependent manner, thus suggesting that reduced *SLC25A1* allele dosage is indeed pathogenic [15,61,62]. However, importantly, 22q11.2 microdeletion syndrome is survivable and affected individuals can have long life expectancy when severe heart abnormalities do not complicate the disease.

Various *SLC25A1* missense or truncating mutations spanning throughout the coding region have been reported as either homozygous or compound heterozygous in a heterogeneous group of developmental disorders and in D-2-/L-2-hydroxyglutaric aciduria [5,63,64,65,66,67,68]. Some of these compound mutations lead to a more complete and severe loss of CIC activity relative to 22.q11.2 deletion syndromes and are accompanied by a broader clinical spectrum of manifestations, which includes various craniofacial abnormalities (facial dysmorphism and macro/microcephaly), brain abnormalities, epilepsy, respiratory insufficiency and encephalopathy. A variable degree of metabolic dysfunction is seen in the affected individuals, represented by lactic acidosis, urinary excretion of TCA cycle intermediates (fumarate, succinate and α-KG) as well as of two metabolites, D2-L2 hydroxyglutaric acids (D-L-2HG). In addition, defects in respiratory complex subunits have been occasionally described in patients harboring *SLC25A1* gene mutations [67]. This clinical spectrum of manifestations together with lactic acidosis and the alterations of the TCA cycle are hallmarks of mitochondrial dysfunction and point to the possibility that diseases sustained by CIC deficiency should be re-classified as mitochondrial disorders.

**Figure 8:**
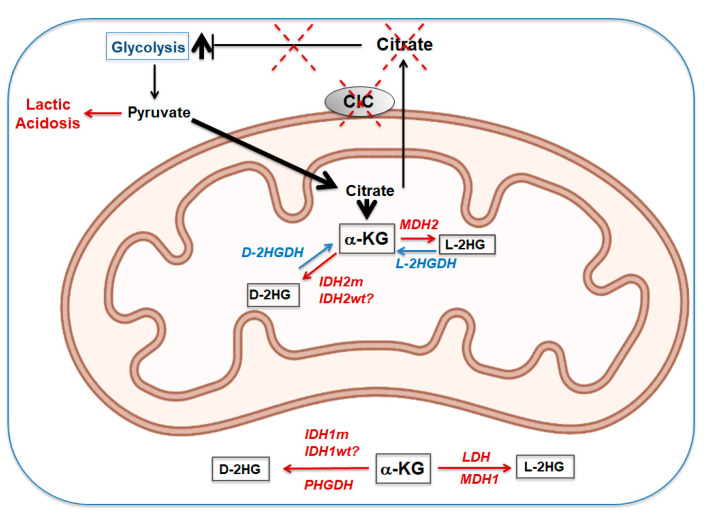
Mitochondrial and cytosolic pathways leading to D2-L2 hydroxyglutaric acid (D-L-2HG) accumulation (see text for explanation). The proposed model envisions that as a consequence of disruption of CIC-mediated citrate export activity, cytosolic citrate is reduced, leading to loss of the feedback loop on PFK. This leads to enhanced glycolysis and production of pyruvate, which, on one side, is converted to lactate, resulting in lactic acidosis. Excess pyruvate also enters mitochondria, where it is converted to citrate/isocitrate. Due to a lack of the export activity of CIC, this excess citrate is converted to TCA cycle intermediates downstream of citrate, leading to accumulation of α-KG and also of succinate, fumarate and malate (not shown in the figure), which are then secreted in urine. In red are the potential steps for conversion of α-KG to L-2HG or D-2HG, by either IDH1 or IDH2; in blue are the enzymes involved in the degradation pathway. IDH1/IDH2m or wt: mutant or wild-type forms of these enzymes. See text for additional abbreviations.

Whilst the accumulation of TCA cycle intermediates downstream of citrate/isocitrate can be explained by the lack of the only mechanism of export of citrate away from the mitochondria, namely CIC (Figure 8), an important question in the context of these disorders regards both the origin and the significance of the D-2-/L-2-hydroxyglutaric aciduria (D-L-2HGA).

Individual L- or D-2HGAs are severe developmental disorders caused by mutations in L2-hydroxyglutarate dehydrogenase (*L2HGDH*) or D-2-hydroxyglutarate dehydrogenase (*D2HGDH*) that eliminate L2- or D2-HG, respectively. Neomorphic mutations of *IDH2* that convert α-KG to D-2HG can also cause 2HGA [69,70]. However, combined D-L-2-HGA is only sustained by *SLC25A1* gene mutations, with an apparent preference for D-2HG accumulation in all body fluids. Combined L-D-2HGA was already suspected as a distinct clinical entity by the Jakobs group before alterations of the *SLC25A1* gene were discovered as the cause of the disease [71].

The first attempt to classify these disorders came from the seminal work of the Salomons group that retrospectively examined a number of patients presenting with various degrees of pathological manifestations [72], reviewed in [5]. The most important outcome of this analysis is that there is a clear correlation between the extent of loss of the citrate export activity of CIC—which differs depending upon the affected amino acid—and the severity of the clinical course, as well as life expectancy. Noticeably, the most severe phenotype is seen with the compound heterozygous mutations p.A9Profs*82 and p.P45L, which have been described in several affected patients. p.A9Profs*82 results in a truncated protein, while P45 maps to the region involved in mitochondrial translocation, likely displacing CIC from the mitochondria and resulting in more severe complete loss of mitochondrial activities compared to other mutations. This allele combination is likely to recapitulate a nearly complete null phenotype. Children carrying these mutations display the most severe and earliest onset spectrum of clinical manifestations, culminating in early death [65,66]. These comprise craniofacial abnormalities and facial dysmorphism, microcephaly as well as hallmarks of mitochondrial dysfunction including encephalopathy, myopathy, respiratory insufficiency, lactic acidosis and accumulation of TCA cycle intermediates. However, whether these clinical manifestations are actually connected to accumulation of L-D-2HGs is still not entirely clear. For example, in two siblings harboring the p.A9Profs*82 and p.P45L alleles, only one displayed very high levels of 2-HGs, while in serial measurements performed in the second sibling, 2-HGs levels were near-normal or moderately elevated, yet the clinical course was similarly devastating in both patients [65]. In other instances of *SLC25A1* gene mutations, the levels of L-D-2HGs have been reported either as very high or only moderately or inconsistently increased. Therefore, an important question is whether in some situations, accumulation of 2-HGs is an innocent bystander alteration of CIC inactivation, while in others, the contribution of additional factors exacerbates the extent of 2-HGA and the associated pathology. Development of molecular biomarkers for 2HG activities (e.g., alterations in methylation patterns) is also needed to overcome the shortcomings of snapshot measurements of these metabolites in hardly accessible biological fluids (e.g., cerebrospinal fluid or intracellular space).

The reason why cells where CIC is inactive accumulate 2HGs is also not entirely clear (see Figure 6). Wild-type IDH1 and IDH2 have been reported to produce low levels of 2-HGs under reductive carboxylation conditions [73], raising the possibility that this mechanism comes into play in these disorders. Alternative sources of 2-HGs are the promiscuous activities of malate or lactate dehydrogenase (MDH1/2 and LDH, respectively), impaired activity of L2- or D2-HGDHs due to excess substrate(s) or altered redox balance [69,74,75]. Reduced concentrations of malate and increased levels of NADH, hypoxia or metabolic acidosis due to enhanced lactic acid concentrations can all favor the promiscuous activity of MDH2 or LDH [73]. Furthermore, 3-phosphoglycerate dehydrogenase (PHGDH) can also catalyze the NADH-dependent reduction of α-KG to D-2HG [76].

It is noticeable that all these routes for the accumulation of 2-HGs involve α-KG, and therefore, understanding and targeting the pathways leading to α-KG accumulation or the enzymes involved in its conversion to 2-HGs should provide important strategies to ameliorate L-D-2HGA. Based on these considerations, here, we pose several questions, the answer to which, we believe, should advance this field forward:

1. What levels of L-D-2HG in the context of *SLC25A1* gene deficiency are to be considered pathogenic?
2. Given that L- or D-HGs interfere with α-KG-dependent dioxygenases and affect methylation of histones and DNA, are alterations in L-D-2HG levels reflected in changes in the epigenetic landscape of CIC-deficient cells?
3. What is the origin of L-D-2HG accumulation in CIC-deficient cells?
4. Can correction of α-KG levels normalize any of the pathological manifestations seen in children with CIC deficiency?
5. Are there differences in the lipid synthetic pathway in diseases sustained by CIC deficiency that aggravate the clinical manifestations?
6. Is the multi-organ involvement seen in severe CIC deficiency syndromes due to systemic alterations (e.g., changes in circulating levels of TCA cycle intermediates, accumulation of toxic byproducts of the metabolism and elevated levels of 2-HGs) or to loss of tissue-specific activities of CIC?

Though many of the pathological manifestations due to CIC deficiency are acquired during embryogenesis and development and, therefore, are likely irreversible, the answers to these questions could lead to the development of therapeutic strategies able to ameliorate at least some aspects of these devastating disorders.
